# Single-cell analysis of anchorage-independent growth ability in pancreatic ductal adenocarcinoma cell lines

**DOI:** 10.1186/s13104-026-07670-4

**Published:** 2026-01-24

**Authors:** Yuuki Shichi, Seiichi Shinji, Masakazu Fujiwara, Yutaro Ogawa, Yusuke Yoshimura, Keisuke Nonaka, Hiroshi Yoshida, Toshiyuki Ishiwata

**Affiliations:** 1 Division of Aging and Carcinogenesis, Research Team for Geriatric Pathology, Tokyo Metropolitan Institute for Geriatrics and Gerontology, Tokyo, 173-0015 Japan; 2 Department of Gastroenterological Surgery, Nippon Medical School, 1-1-5 Sendagi, Bunkyo-ku, Tokyo, 113-8603 Japan

**Keywords:** Pancreatic ductal adenocarcinoma, 3D culture, Sphere, Single-cell analysis, Anchorage-independent growth

## Abstract

**Objective:**

Anchorage-independent growth is a critical feature of cancer cells, reflecting their ability to survive and proliferate without attachment to the extracellular matrix. Spheres—cancerous masses formed in a three-dimensional (3D) anchorage-independent culture—contain a high level of cancer stem cells. This anchorage-independent proliferative capacity closely relates to tumorigenicity, anoikis resistance, and metastatic capability. Pancreatic ductal adenocarcinoma (PDAC) is a heterogeneous group comprising epithelial and mesenchymal features, and these subtypes exhibit different biological characteristics in 3D cultures. This study examines whether these PDAC subtypes differ in their anchorage-independent proliferative capability at the single-cell level.

**Methods:**

Eight PDAC cell lines, including five epithelial-type and three mesenchymal-type lines, were cultured as single cells in poly (2-methacryloyloxyethyl phosphorylcholine) (MPC) polymer–coated low-attachment microwell plates, and time-lapse imaging was performed every 15 min for 60 h.

**Results:**

Three phenotypes were observed: non-proliferating single cells, cells dividing into two, and those forming clusters of three or four cells. In single-cell analysis, KP4 and MIA PaCa-2 mesenchymal PDAC cells exhibited a high number of cells proliferating into two or more cells.

**Conclusion:**

These findings suggest that mesenchymal PDAC cells exhibit greater anchorage-independent proliferative capability, reflecting their aggressive biological behavior.

**Supplementary Information:**

The online version contains supplementary material available at 10.1186/s13104-026-07670-4.

## Introduction

Normal epithelial cells require attachment to the extracellular matrix or a culture plate’s surface for survival and proliferation [1], a phenomenon known as anchorage-dependent growth. When deprived of such attachment, these cells undergo a specific form of apoptosis known as anoikis [2]. Conversely, cancer cells typically lose this anchorage dependence, enabling them to grow in suspension, thereby forming multicellular aggregates known as spheres or spheroids within soft agar or liquid culture conditions [3]. Recent studies have indicated that anchorage-independent spheres are often enriched in cancer stem cells (CSCs), which exhibit self-renewal and multipotent differentiation capabilities [4]. CSCs are believed to contribute to tumor heterogeneity, metastasis, and resistance to conventional therapies [5].

Pancreatic ductal adenocarcinoma (PDAC) remains one of the most lethal and treatment-refractory malignancies. With the aging population, its incidence and mortality are expected to increase. PDAC is expected to become the second leading cause of cancer-related deaths in the U.S. by 2030 [6]. Notably, its cell lines have been reported to exhibit distinct molecular phenotypes, with some expressing epithelial marker mRNAs and others expressing mesenchymal marker mRNAs [7]. In our recent study, we indicated that when cultured under low-attachment conditions, PDAC cell lines form floating spheres with distinct morphological characteristics depending on whether the cells exhibit epithelial or mesenchymal traits [8, 9]. These differences between epithelial and mesenchymal features are associated with PDAC cell proliferation, migratory behavior, and drug sensitivity [10, 11]. However, it remains unclear whether the morphological and functional differences observed in spheres arise from the intrinsic properties of individual cancer cells. This is primarily because of the difficulty in culturing and analyzing single cancer cells in a non-adherent manner.

This study employed a novel culture slide system containing up to 90,000 non-adhesive microwells that enable the isolation and culture of individual PDAC cells under anchorage-independent conditions. Using time-lapse imaging, we monitored the proliferation dynamics of single cells derived from epithelial- and mesenchymal-type PDAC cell lines. Subsequently, we quantified the proportion of cells in each line that underwent cell division to form two or more daughter cells.

## Materials and methods

### PDAC cell lines

PK-8, PK-45P, T3M-4, and KP4 human PDAC cells were provided by the RIKEN BioResource Research Center through the National Bio-Resource Project of the Ministry of Education, Culture, Sports, Science, and Technology of Japan. Additionally, PK-59, PK-1, PANC-1, and MIA PaCa-2 human PDAC cell lines were obtained from the Cell Resource Center for Biomedical Research of the Institute of Development, Aging, and Cancer of Tohoku University (Sendai, Japan). The cells were cultured in RPMI-1640 medium supplemented with 10% fetal bovine serum at 37 °C in a humidified environment containing 5% CO_2_.

### Anchorage-independent growth in single-cell cultures

To evaluate the proliferative capacity of PDAC cells at the single-cell level under anchorage-independent conditions, PDAC cells were seeded at 3.0 × 10^3^ cells in Sievewell (Tokyo Ohka Kogyo, Co. Ltd. Kanagawa, Japan). The 50 μm type of Sievewell is a slide glass-sized chamber containing 90,000 wells, each with a diameter and depth of 50 μm (Supplemental Fig. S1). The bottom and surrounding surfaces of the microwells are coated with poly (2-methacryloyloxyethyl phosphorylcholine) (MPC polymer), creating a low-adhesion surface that prevents cell attachment. After applying the PDAC cells to the Sievewell, the medium is aspirated through a side port using a pipette, causing the medium to flow out through the holes at the bottom of each well, following the manufacturer’s instructions. This process distributes individual PDAC cells into adjacent wells, achieving one cell per well. Although the PDAC cells share the same medium, the well walls physically isolate them, enabling single-cell culture under low adhesion states without direct cell-to-cell contact.

### Time-lapse imaging acquisition

Time-lapse images of the process of PDAC cell proliferation in the low-attachment microwells were administered with a BZ-X710 fluorescence microscope (Keyence, Osaka, Japan) for 60 h at 15-min intervals for each cell. A 10x magnification objective lens was used to capture the cell proliferation process. In the time-lapse images, we first confirmed PDAC in the microwells and observed cell proliferation in the wells for 60 h. A cell was excluded from analysis if it migrated out of the microwell or if another cell entered the well, because these conditions prevent accurate single-cell tracking. We conducted three biologically independent experiments. In total, each PDAC cell line included the analysis of at least 280 single cells obtained from a minimum of 12 microscopic fields. Subsequently, the PDAC cells were classified as (1) cells that did not proliferate, (2) cells that proliferated to two, and (3) cells that proliferated to three or more (Supplemental Fig. S1).

### Statistical analysis

Statistical analyses were performed using GraphPad Prism version 10.6.1 (GraphPad Software, San Diego, CA, USA). Single-cell division events were treated as ordinal variables (0–3 divisions). Differences among the eight PDAC cell lines were evaluated using the Kruskal-Wallis test followed by Dunn’s multiple comparisons test with a familywise α = 0.05. Adjusted p-values were used to determine statistical significance. Group-wise significance in the figures is summarized using compact letter display, and the complete list of pairwise adjusted p-values is provided in Supplementary Table S2.

## Results

### Anchorage-independent proliferative capacity of epithelial PDAC cell lines

**Fig. 1:**
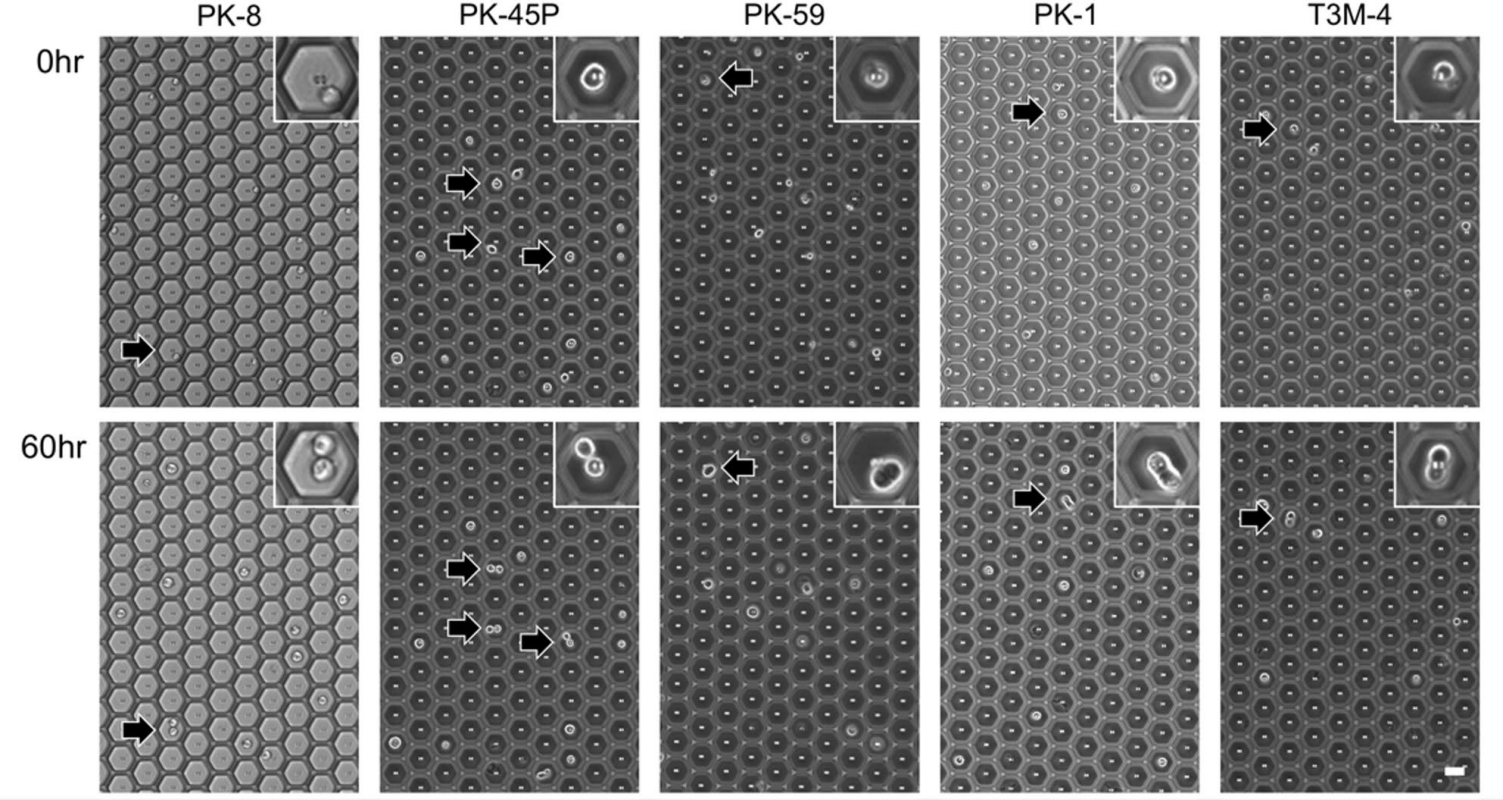
Characteristic mitotic figures of epithelial PDAC cells in microwells. Five epithelial PDAC cell lines were seeded in a microwell plate. The top row shows images of the cells at zero hours, and the bottom row shows the same cells at 60 h. Arrows indicate epithelial PDAC cells that have proliferated into two. The inset is an enlarged image of the arrowed area. Scale bars = 50 μm

**Fig. 2:**
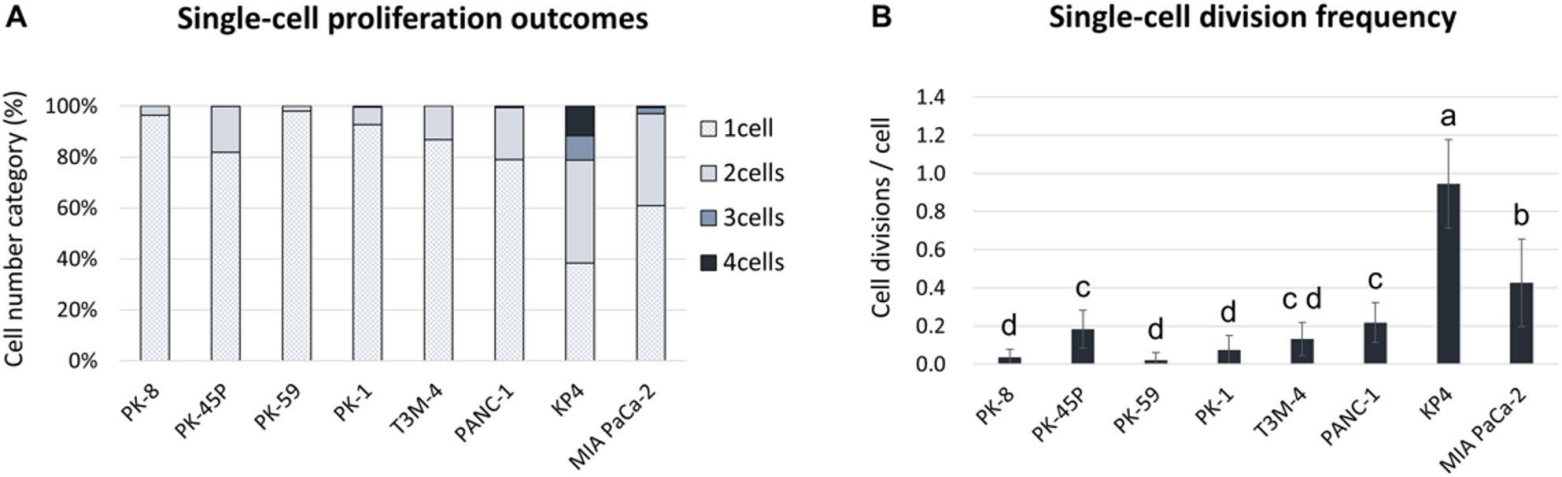
Single-cell proliferation analysis of eight PDAC cell lines cultured in low-adhesion microwells for 60 h. **A** Proportional distribution (%) of final cell numbers (1–4 cells) obtained from single PDAC cells after 60 h of time-lapse tracking. **B** Mean ± SD of division events (0–3) per single cell over 60 h. Statistical comparisons were performed using the Kruskal–Wallis test followed by Dunn’s multiple comparisons test. Compact letters indicate statistically homogeneous groups; cell lines not sharing a letter differ significantly. Full adjusted p-values are provided in Supplementary Table S2

Epithelial PDAC cell lines, such as PK-8, PK-45P, PK-59, PK-1, and T3M-4 cells [8], were observed to proliferate in two during 60 h (Fig. 1, arrows and insets). In these cell lines, less than 20% of all cells proliferated in two, with PK-45P cells being the most common; cells proliferating in three or four were observed only in 0.2% of PK-45P and 0.3% of PK-1 cells; and no other PDAC cell lines showed proliferation in three or more (Fig. 2, Supplemental Table S1). In addition to proliferating cells, we quantified the proportion of single cells that did not increase in number during the 60-h observation period. Across epithelial PDAC cell lines, 81.8–97.9% of seeded single cells failed to divide (Fig. 2, Supplemental Table S1). These non-proliferating cells were included in our analysis because they may represent cells undergoing anoikis or entering a dormant state.

### Anchorage-independent proliferative capacity of mesenchymal PDAC cell lines

**Fig. 3:**
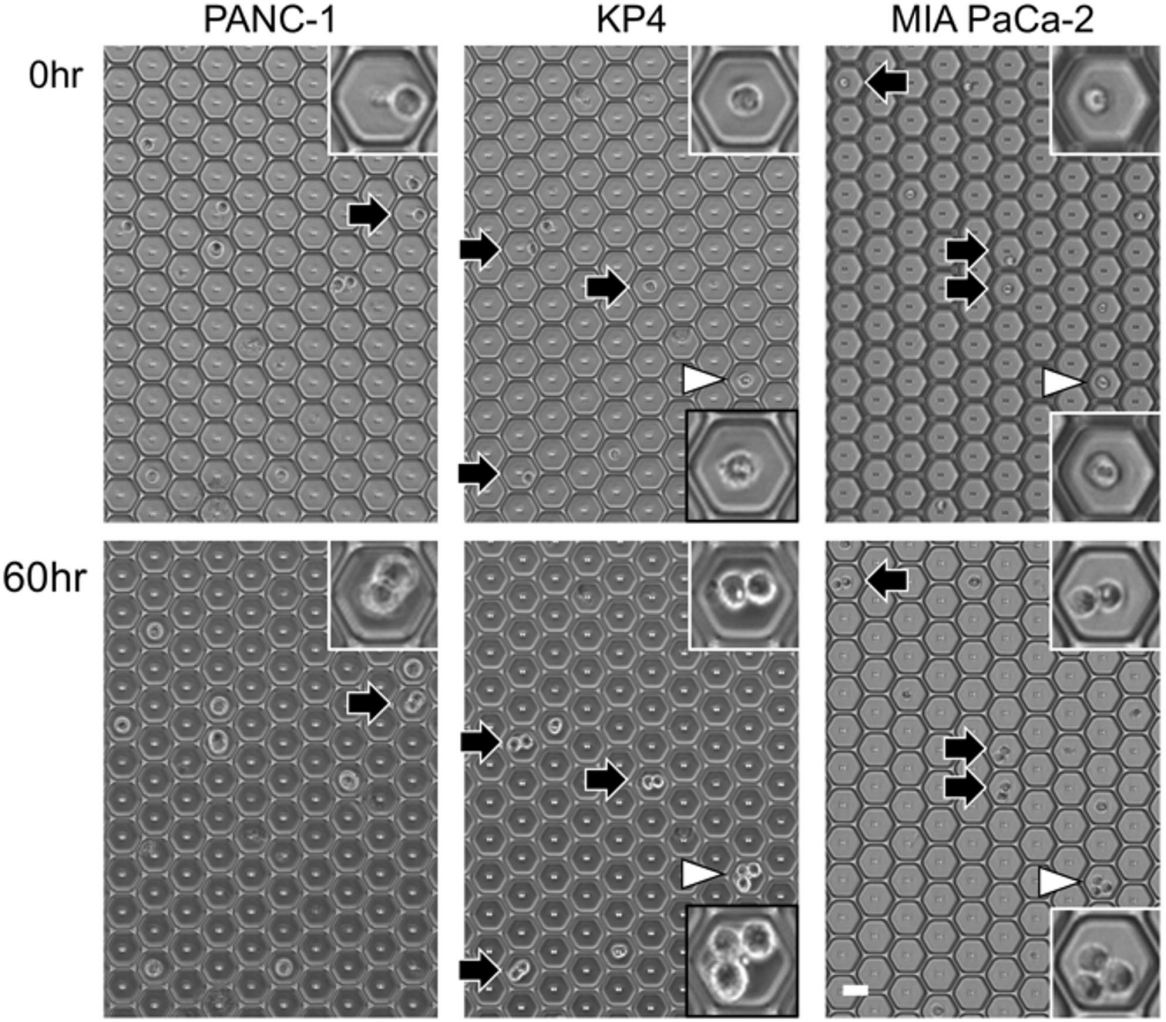
Characteristic mitotic figures of mesenchymal PDAC cells in microwells. Three mesenchymal PDAC cell lines were seeded in a microwell plate. The top row shows images of the cells at zero hours, and the bottom row shows the same cells at 60 h. The arrows indicate mesenchymal PDAC cells that have proliferated into two cells, and the arrowheads indicate mesenchymal PDAC cells that have proliferated into three cells. The inset is an enlarged image of the arrowed area. The inset in the black frame is an enlarged image of a cell that has divided into three cells. Scale bars = 50 μm

In the mesenchymal PDAC cell line, cells that proliferated to two and those that proliferated to three or four were found (Fig. 3, arrow and arrowheads, respectively, in insets). The mesenchymal PDAC cell line KP4 cells had the most proliferating cells with 61.7% of the cells proliferating (Fig. 3, Supplemental Table S1). Approximately 35% of these proliferated cells had proliferated into three or four cells. The mesenchymal cell lines KP4 and MIA PaCa-2 cells had more proliferating cells than the epithelial PDAC cell lines (Fig. 2A, B).

## Discussion

In recent years, increasing attention has been paid to the intratumoral heterogeneity in cancer, not only among patients but also within the same tumors [12–14]. However, analyzing the proliferative capacity of large numbers of cancer cells at the single-cell level remains technically challenging, and few studies have addressed this issue.

In this study, we utilized a micro-scale low-adhesion culture well system that isolates single PDAC cells into each microwell. Although not all wells contained a single cell upon seeding, wells containing two or more cells were rare. To accurately assess whether single PDAC cells could proliferate into two or more daughter cells, we performed continuous time-lapse imaging for up to 60 h immediately after seeding. Only wells that initially contained one cell and that did not receive any migrating cells from neighboring wells during the observation period were included in the analysis. This approach enabled us to quantitatively evaluate the anchorage-independent proliferation capacity of epithelial-type and mesenchymal-type PDAC cell lines at the single-cell level.

In addition to previously established single-cell suspension and sphere-formation systems, our microwell-based platform offers a complementary approach optimized for the quantitative evaluation of anchorage-independent growth in PDAC. Several prior methods, including high-throughput single-cell–derived sphere assays [15] and prostate cancer stem/progenitor sphere-formation protocols [16], have successfully enabled clonal sphere growth in low-adhesion environments. Moreover, live single-cell tracking approaches have been used to predict sphere-forming capacity from early divisions in breast cancer cells [17]. While these systems demonstrate an excellent capability for monitoring stemness-associated sphere formation, they were not designed to dissect the anchorage-independent proliferation behavior that is specific to PDAC.

In contrast, our method focuses on characterizing single-cell dynamics in PDAC under strictly non-adherent microwell conditions created by MPC-polymer coating and integrates long-term time-lapse imaging to rigorously confirm cell non-adhesion and to track division patterns. Our approach therefore does not aim to introduce a new culturing technology but rather to leverage a well-validated microwell system to characterize PDAC cell-type–dependent heterogeneity in anchorage-independent growth, which has not been previously addressed.

Cell cycle period for normal cells and PDAC cells has been reported as 24–48 h [18]. During the 60-hour observation period, a significant proportion of single PDAC cells did not divide, suggesting that these non-dividing cells may have entered a dormant state outside the active cell cycle. However, some cells appeared to divide once or twice during the 60-hour observation period, consistent with previous reports [18]. Our previous studies demonstrated that mesenchymal PDAC cell lines form spheres with a higher proportion of Ki-67-positive proliferative cells in three-dimensional (3D) culture and exhibit increased viability as measured by ATP assays compared to epithelial PDAC cell lines [8]. Moreover, when spheres were re-plated onto adhesive surfaces, mesenchymal PDAC cells exhibited faster and more extensive migration compared to epithelial PDAC cells [11]. Regarding drug sensitivity, gemcitabine was more effective in epithelial PDAC cell lines, while nab-paclitaxel showed greater efficacy in mesenchymal PDAC cell lines in 3D culture [8]. These findings align with the current observation that KP4 and MIA PaCa-2 cells—both mesenchymal-type lines—exhibited higher proliferative capacity at the single-cell level under low-attachment conditions. Many epithelial cancer cells are known to undergo anoikis when placed in non-adherent conditions. In our dataset, the substantial fraction of single cells that did not proliferate during the 60-h window likely includes cells experiencing anoikis, as well as those entering a transient dormant state, as described in previous reports [19–21]. Although we did not perform additional apoptosis or anoikis-specific staining, the quantification of non-proliferating cells provides indirect evidence consistent with these processes.

In some PDAC cell lines, a single cell increased to three cells within 60 h. Although detailed morphological evaluation was difficult at the magnification and resolution used in this study, these observations suggest the possibility of asymmetric cell division. CSC may continuously produce differentiated cancer cells. In the future, utilizing single-cell RNA sequencing may help elucidate the possible connection between these anchorage-independent proliferative PDAC cells and CSC. Although non-standard increases in cell numbers were observed, these data alone are insufficient to infer asymmetric division. We therefore interpret them conservatively as irregular division patterns.

In this study, we focused primarily on the phenotypic characterization of anchorage-independent proliferation at the single-cell level. To further investigate the underlying biological mechanisms, we attempted single-cell qRT-PCR analysis of CSC and EMT markers. However, because the RNA yield from individual PDAC cells within each microwell was extremely low, we were unable to obtain sufficient RNA for reliable gene expression analysis. Due to these technical limitations, mechanistic validation could not be included in the present study. Clarifying the molecular features of anchorage-independent proliferative PDAC cells therefore remains an important future direction for understanding PDAC heterogeneity.

Although our microwell system enabled the precise tracking of single PDAC cells under non-adherent conditions, several limitations should be noted. The irregular increases in cell number (e.g., 1→3 or 1→4) do not provide sufficient evidence for asymmetric division, and we therefore interpreted them conservatively as non-standard proliferative patterns. Moreover, because no anoikis-specific staining was performed, non-proliferating cells cannot be definitively classified as undergoing anoikis. These points should be considered when interpreting our findings.

In conclusion, this experimental method was useful for analyzing the proliferation behavior of single PDAC cells under non-adhesive conditions. The results revealed that mesenchymal PDAC cell lines have higher proliferative capacity than epithelial PDAC cell lines, even at the single-cell level. This single-cell analysis method is expected to play a significant role in elucidating the functions and therapeutic responsiveness of individual cells within heterogeneous PDAC populations.

## Supplementary Information

Supplementary Material 1.

Supplementary Material 2.

Supplementary Material 3.

Supplementary Material 4.

Supplementary Material 5.

Supplementary Material 6.

Supplementary Material 7.

Supplementary Material 8.

Supplementary Material 9.

## Data Availability

Data supporting the findings of this study are available from the corresponding author upon reasonable request.

## Abbreviations

3D: Three-dimensional
PDAC: Pancreatic ductal adenocarcinoma
CSCs: Cancer stem cells
