# Supplementary material for: Single-cell analysis of anchorage-independent growth ability in pancreatic ductal adenocarcinoma cell lines
Source: BMC Res Notes. 2026 Jan 24;19:80. doi: 10.1186/s13104-026-07670-4 (PMC12911202; doi:10.1186/s13104-026-07670-4)
Supplement: Supplementary file 1 — Supplementary Material 1. [file 13104_2026_7670_MOESM1_ESM.docx]

**Supplemental materials**

**Single-cell analysis of anchorage-independent growth ability in pancreatic ductal adenocarcinoma cell lines**

Yuuki Shichi^a^†, Seiichi Shinji^a,b^†, Masakazu Fujiwara^a^, Yutaro Ogawa^a,b^, Yusuke Yoshimura^a,b^, Keisuke Nonaka^a^, Hiroshi Yoshida^b^, Toshiyuki Ishiwata^a*^

^a^ Division of Aging and Carcinogenesis, Research Team for Geriatric Pathology, Tokyo Metropolitan Institute for Geriatrics and Gerontology, Tokyo 173-0015, Japan, y_shichi@tmig.or.jp; mfujiwa@tmig.or.jp; nona_kei@tmig.or.jp; tishiwat@tmig.or.jp

^b^ Department of Gastroenterological Surgery, Nippon Medical School, 1-1-5 Sendagi, Bunkyo-ku, Tokyo 113-8603, Japan, s-shinji@nms.ac.jp; s11-024oy@nms.ac.jp; s13-110yy@nms.ac.jp; hiroshiy@nms.ac.jp

†These authors contributed equally to this work.

**^*^**Corresponding author: Toshiyuki Ishiwata

35-2 Sakae-cho, Itabashi-ku, Tokyo, 173-0015 Japan

Phone: +81-3-3964-3241 ext. 4414. Fax: +81-3-3579-4776

E-mail: tishiwat@tmig.or.jp

**Supplemental Fig. S1**

**Pancreatic ductal adenocarcinoma (PDAC) cells cultured as**
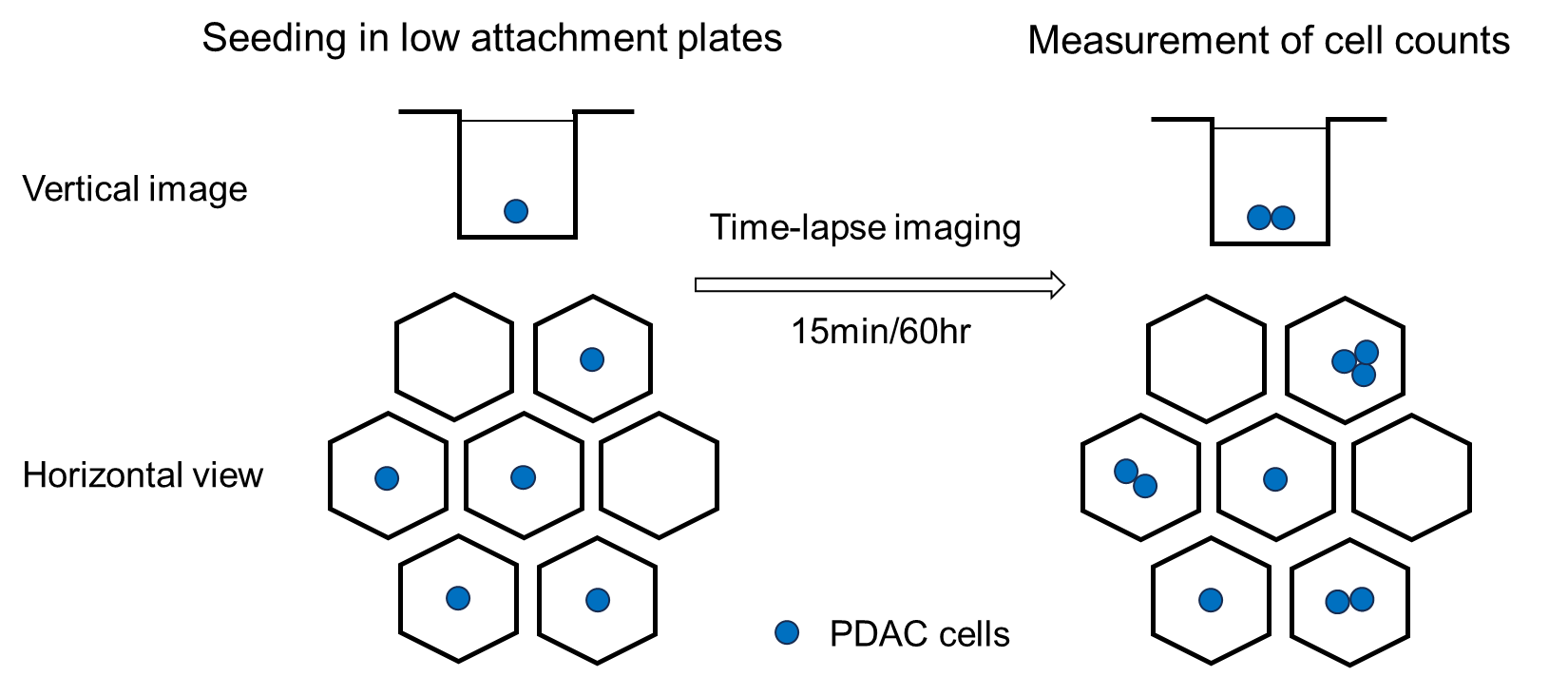
**single cells in low-attachment microwells.**

A single PDAC cell was cultured in a low-adhesion microwell with a hexagonal shape and a diameter of 50 μm, photographed every 15 minutes for 60 hours, and the number of cells that increased to two or more was counted.

**Supplemental Table S1**

**Number of divided pancreatic ductal adenocarcinoma (PDAC) cells cultured as single cells in low-adhesion micro wells**

| **Cell Line** | **1 Cell (%)** | **2 Cells (%)** | **3 Cells (%)** | **4 Cells (%)** |
| --- | --- | --- | --- | --- |
| PK-8 | 96.4 ± 4.2 | 3.6 ± 4.2 | – | – |
| PK-45P | 81.8 ± 9.9 | 18.0 ± 9.9 | 0.2 ± 0.7 | – |
| PK-59 | 97.9 ± 3.9 | 2.1 ± 3.9 | – | – |
| PK-1 | 92.8 ± 6.9 | 6.9 ± 6.7 | 0.3 ± 1.6 | – |
| T3M-4 | 86.9 ± 8.8 | 13.1 ± 8.8 | – | – |
| PANC-1 | 79.0 ± 10.1 | 20.4 ± 10.3 | 0.3 ± 1.1 | 0.3 ± 1.3 |
| KP4 | 38.4 ± 12.3 | 40.4 ± 9.4 | 9.6 ± 7.0 | 11.7 ± 7.0 |
| MIA PaCa-2 | 61.0 ± 18.5 | 36.0 ± 16.6 | 2.4 ± 4.1 | 0.6 ± 2.1 |

A single PDAC cell was cultured in a low-adhesion microwell with a hexagonal shape and a diameter of 50 μm for 60 hours. Time-lapse imaging was performed every 15 minutes, and the number of cells in the well was measured after 60 hours. Values represent mean ± SD from three independent experiments. “–” indicates no observed events.

**Supplemental Table S2**

**Results of Dunn’s multiple comparisons test for division events among eight PDAC cell lines**

| Comparison | Mean rank difference | Adjusted p-value | Significance |
| --- | --- | --- | --- |
| PK-8 vs. PK-45P | -191.1 | <0.001 | *** |
| PK-8 vs. PK-59 | 10.02 | >0.99 | ns |
| PK-8 vs. PK-1 | -49.55 | >0.99 | ns |
| PK-8 vs. T3M-4 | -124.2 | 0.15 | ns |
| PK-8 vs. PANC-1 | -212.9 | <0.001 | *** |
| PK-8 vs. KP4 | -818.9 | <0.001 | *** |
| PK-8 vs. MIA PaCa-2 | -451.6 | <0.001 | *** |
| PK-45P vs. PK-59 | 201.1 | <0.001 | *** |
| PK-45P vs. PK-1 | 141.5 | 0.02 | * |
| PK-45P vs. T3M-4 | 66.83 | >0.99 | ns |
| PK-45P vs. PANC-1 | -21.86 | >0.99 | ns |
| PK-45P vs. KP4 | -627.8 | <0.001 | *** |
| PK-45P vs. MIA PaCa-2 | -260.5 | <0.001 | *** |
| PK-59 vs. PK-1 | -59.56 | >0.99 | ns |
| PK-59 vs. T3M-4 | -134.3 | 0.15 | ns |
| PK-59 vs. PANC-1 | -223 | <0.001 | *** |
| PK-59 vs. KP4 | -828.9 | <0.001 | *** |
| PK-59 vs. MIA PaCa-2 | -461.6 | <0.001 | *** |
| PK-1 vs. T3M-4 | -74.7 | >0.99 | ns |
| PK-1 vs. PANC-1 | -163.4 | 0.01 | * |
| PK-1 vs. KP4 | -769.3 | <0.001 | *** |
| PK-1 vs. MIA PaCa-2 | -402 | <0.001 | *** |
| T3M-4 vs. PANC-1 | -88.69 | >0.99 | ns |
| T3M-4 vs. KP4 | -694.6 | <0.001 | *** |
| T3M-4 vs. MIA PaCa-2 | -327.3 | <0.001 | *** |
| PANC-1 vs. KP4 | -605.9 | <0.001 | *** |
| PANC-1 vs. MIA PaCa-2 | -238.6 | <0.001 | *** |
| KP4 vs. MIA PaCa-2 | 367.3 | <0.001 | *** |
